# The Effectiveness of the Moving to Emptiness Technique on Clients Who Need Help During the COVID-19 Pandemic: A Real-World Study

**DOI:** 10.3389/fpubh.2022.890960

**Published:** 2022-05-18

**Authors:** Yanqiang Tao, Yi Chen, Wen Zhou, Lihui Lai, Tianjun Liu

**Affiliations:** ^1^Beijing Key Laboratory of Applied Experimental Psychology, School of Psychology, Beijing Normal University, Beijing, China; ^2^School of Psychology, Nanjing Normal University, Nanjing, China; ^3^Yikong Skill Research Institute, Nanjing, China; ^4^School of Continuing Education, Guangzhou University, Guangzhou, China; ^5^School of Acupuncture-Moxibustion and Tuina, Beijing University of Chinese Medicine, Beijing, China

**Keywords:** moving to emptiness, COVID-19, psychological consultation, clients, Chinese traditional cultural

## Abstract

With Western therapeutic techniques prevailing in Chinese therapies, some techniques that include Chinese traditional cultural features are required since some cultural factors are not considered in the Western method. Our study introduced a new technique, the moving to emptiness technique (MET), which combines Western structural progress and core factors of Chinese culture. Seventeen therapists treated 107 clients with the MET. Clients reported their target symptoms initially, and therapists helped them transfer invisible symptoms to perceivable stuff and remove their jarring stuff using the psychological emptiness area. At the end of the consultations, we found that MET could eliminate symptoms immediately. By grouping target symptoms according to their frequency, the results showed that clients in the high-frequency symptom group had higher rehabilitation rates than those in the low-frequency symptom group. Additionally, the results of the bereavement group were better than those of the non-bereavement group, indicating that the MET can significantly alleviate clients' target symptoms. In future studies, the replication and stability of the MET can be assessed by integrating questionnaires, experimental designs, and neurological equipment.

“There is no Bodhi tree, mirror, or stand. Originally, there was nothing around us. Therefore, no dust will fall.” – Hui Neng, the founder of Zen Buddhism.

## Introduction

There are many psychosomatic symptoms in the community as a result of the COVID-19 pandemic, such as panic, tension, melancholy, and helplessness. Therefore, China has paid increasing attention to providing psychological therapy for those with mental health problems. However, most therapists in China still use Western therapies, which leads to some cultural maladjustment. Because of this, some experts in China are now researching the application of Chinese traditional philosophy and culture in Western structural therapies. The moving to emptiness technique (MET) has become a choice for therapists in this context.

## The Theoretical Foundation of the MET

In one branch of Chinese philosophy, nothing exists around us. Anything that disturbs us comes from our inner world. Therefore, if we put any of our problems into a more extensive context, the so-called emptiness state, our mental troubles will no longer exist. Clinical psychology is regarded as a valuable field for all human beings in China, even if the research method is still new. Moreover, China has a long history of psychological thinking, which can be traced back to various traditional philosophical and medical works (1, 2). Confucianism, Taoism, and Buddhism are three philosophical branches that impact Chinese civilization on a large scale. As mentioned earlier, poems are common in modern China, in which principles lay a solid foundation for Chinese people's faith and actions. Additionally, these philosophical ideas have influenced the Chinese medical system since ancient times, especially in treating mental disorders and illnesses and cultivating a healthy life. The MET is one therapeutic skill that applies the essence of traditional Chinese philosophical thinking and cultivates medical systems in clinical therapies (3).

Structural therapeutic techniques, such as cognitive behavioral therapy, [CBT, (4)] and non-structural therapeutic techniques, such as psychoanalysis, are two main methods that are popular among therapists. CBT is a type of therapy in which therapists focus on how clients' dysfunctional beliefs affect their current behaviors and functions (5). CBT helps clients explore, challenge, and modify their dysfunctional beliefs, which is called cognitive restructuring, and can transfer the distorted way they interpret reality into a more adaptive direction. CBT offers an operational structure for therapists to lead clients in identifying their beliefs and core values and then better revise them by considering new possibilities (6).

Therapists who apply CBT assign homework outside of therapeutic sessions for clients to experience the value of the proposed changes that were developed through the collaboration between therapists and clients in therapeutic sessions. Instead of revising and restructuring the cognitive system or locus of attention, the MET aims to eliminate negative feelings represented by target symptoms. Taking the structure of CBT into account, the MET is a psychosomatic treatment that includes the academic concept of Chinese medicine in which the priority in treating a mental disorder is to heal the mind. In summary, the MET integrates traditional Chinese culture while preserving somatic relaxation and the operational process of CBT.

In 2019 and 2021, the MET operational manual was published in Chinese (7) and German, respectively. The MET goes beyond structural therapy abilities and includes attempts to discover clients' target symptoms to reduce the symptoms in a broader psychological context. Before therapeutic sessions, therapists help clients relax and lead them in being mindful of their emotions. Following this, the therapists guide the clients to identify and express their target symptoms and place them in appropriate “containers”. With guidance, the clients can move these containers back and forth before their psychological symptoms occur, and then put them even further away until they disappear in a psychological emptiness area. Using ten operational steps (see Section Measures Consultation Step), the MET, a psychosomatic therapy, can reduce or eliminate symptoms.

Compared to Western psychotherapies that focus on “existence,” Chinese therapeutic skills involve both “emptiness” and “existence.” “Emptiness” is a status in which only consciousness exists. At the same time, the existence mentioned here refers to transforming one mood into another or surmounting one without removing anything; for example, transforming a negative mood into a positive mood or overcoming sickness through wellness. In comparison, “emptiness” here relates to psychological emptiness. When a client in a bad mood enters the consultant room, a therapist who uses CBT will guide him or her to identify and restructure the maladaptive cognitive system. Therefore, an unchanged bad mood is covered by a good mood. Once a therapist applies the MET in counseling, the client will be guided to a state of psychological emptiness where the bad mood will disappear. If we describe the so-called state of emptiness in English, it is a neutral, non-positive, and non-negative state of existence instead of a state of action.

The psychological emptiness area can be regarded as a purely mental and emotional condition without any troubles. What must be mentioned here is that clients use their psychological emptiness area to solve their problems, which does not depend on their defensive mechanisms, such as denial, repression, projection, avoidance, transference, replacement, or sublimation. Their troubles are directly absorbed, accepted, and processed rather than being rejected or disguised.

The psychological emptiness area can provide cures because it is an infinite psychological space without any trouble to which therapists can guide clients. Therefore, the problems are put in psychological emptiness, a broader background, which vanish automatically using this skill. For example, if one spoon of salt dissolves in a cup of water, the water will be brackish. Nevertheless, the taste will not change much when the salt is put into one water tank. Moreover, if it is poured into one lake, nothing will change. Theoretically, once a mental disorder can be put into a person's massive psychological emptiness area, it can disappear automatically. In traditional Chinese medicine, the psychological emptiness area is widely proposed for solving psychological troubles (3). The MET provides clear, fast, and practical guidance to enter the psychological emptiness area. It is not only an innovative and non-antagonistic idea but also a particular way for problem solving. Moreover, the entire methodology includes Chinese traditional ideology and wisdom by summarizing the core of the ancient Chinese medical system.

## Goals of Treatment With the MET

As mentioned above, the main difference between the MET and CBT is that the MET directly targets symptom reduction, while CBT focuses more on restructuring the cognitive or emotional systems.

Thus, to test the effectiveness of the MET in practical counseling applications, we sampled 107 subjects to assess whether it could work effectively for the entire group. The index of *influence* was used as the criterion. We first analyzed the immediate and long-term counseling effects of the MET using a paired-samples *t*-test. To specify the effectiveness of the MET for different symptoms, we classified all target symptoms reported into two categories based on a high and low frequency of the top three body parts with the highest presence of symptoms. Since we conducted our research during the COVID-19 pandemic, with clients losing beloved family members, we obtained the clients' familial information before treatment. Based on their backgrounds, we divided them into two groups, the bereavement group or the non-bereavement group, to determine whether the MET has different effects.

## Methods

### Participants and Consultations

#### Participants

We started our study during the COVID-19 pandemic and recruited 107 participants and a total of 17 psychotherapists in mainland China. Notably, some detailed procedures for participants recruitment need to be introduced. Firstly, the present study was an open program to those who require trauma healing treatments due to the COVID-19 pandemic on mainland China and offers them psychological counseling services without requiring a clinical diagnosis at the time of enrollment. However, certain criteria must still be met: experiencing a distressed state of mind during the epidemic, including physical pain such as insomnia, headaches, and chest tightness, as well as negative emotions such as fear, anxiety, guilt, self-blame, irritability, loneliness, and sadness, as well as a desire to improve the psychosomatic condition. Clients in a psychoactive phase or who were unable to complete the three relaxation steps of MET and required crisis assistance were excluded.

We publicized recruitment advertisements on one online platform, and all participants joined voluntarily. The ethics committee of the corresponding author's university approved this research (Reference Number: H20006, ChiCTR2000034164).

### Consultation Steps

#### The Trio (Body, Mind, and Breath) Relaxation Exercise

Regulate the body: Shake and relax the body, sit in the first 1/3 of the chair in a comfortable position with the neck and shoulders relaxed. Straighten the waist and back and then rest both hands on the thighs and close the eyes.

Regulate the breath: Breathe deeply, slowly, and naturally. There is no need to fill or empty the lungs to avoid blood pressure fluctuations.

Regulate the mind: Focus only on exhaling without special attention while inhaling. Empty the mind while exhaling. Practice these steps for ~3 min with the eyes closed and open the eyes when the mind is clear. The relaxation of the body, breath, and mind is the pre-consultation phase and a prerequisite of therapy with the MET. If a client is unable to relax, he or she cannot proceed to the subsequent steps.

#### Select a Symptom That Causes Trouble for the Client as the Target Symptom

It could be a negative emotion such as fear, anxiety, anger, or a negative physical sensation such as tightness of the chest, shortness of breath, or pain of the body. In each session, only one symptom is treated. If there is more than one physical or mental problem, clients will be asked to choose the one that is most problematic or urgent for this session by evaluating its influence on a scale from 0 to 10. Usually, when people seek help, their target symptoms have scores of 7 or above.

#### Visualize and Locate the Target Symptom

There are two ways to determine the symbolic object of the target symptom. One way is to ask the client how they embody their target symptom. For example, the therapist could ask: what makes you feel bad at that body part? Asking such questions could encompass the client's physical sensations and feelings from the target symptom. The other way is to locate the emotion in one somatic part. If the client feels stressed, he or she may report that a pile of cotton is blocking his or her chest to the therapist. After a symbolic object and a certain somatic part are determined and located, the client should elaborate and highlight various dimensions of the object and embody the object. The therapist can help by asking the client to describe the size, shape, weight, sound, texture, and smell related to the symbolic object so it becomes vivid.

#### Visualize a Symbolic Container

A symbolic container is the device that holds that object mentioned in Step 3. It signifies a client's internal resources and energy. Clients are encouraged to create a container with rich and vivid perceptual features that are similar to the symbolic object.

#### Moving the Symbolic Object Into the Psychological Emptiness Area

Guide the client to:

i) put the symbolic object into the container in the mind;
ii) moving the container farther and farther away psychologically and finally into the psychological emptiness area. First, move the container 3 meters away and back again. Repeat this step 2 to 3 times. Then, move the container farther away so that that it will look like a small dot, then move it back. Repeat this process 10 times. Finally, move the container far enough away so the client cannot see or feel it. Clients get to the “emptiness area” where nothing exists in this stage.
iii) When the container with the symbolic object is moved to the psychological emptiness area, clients can feel comfortable and relaxed with the target symptom leaving. The client is asked to experience the sensation of emptiness by staying there as long as they can with their eyes closed.

#### Assessment of Changes After the Intervention

Ask the client to score the influence of their symptoms again after the intervention. If the initial score is reduced by 50% or more, the treatment is considered to be highly effective; if the score is reduced by 1/3 or more but by <50%, the treatment is considered to be adequately effective; and if the score is reduced by <1/3, the treatment is considered to be ineffective.

### Measures

#### Consulting Assessment

A visual analog scale (VAS) is a Likert psychometric scale that is used to evaluate subjective characteristics or attitudes (8). These scales have previously been used to diagnose various disorders in market research and social science assessments.

First, we assessed the influence of the clients' target symptoms and scored it from 1 to 10. At the end of the MET treatment session, the client re-evaluated the impacts of their symptoms when the container was far away and invisible in their minds.

#### Follow-Up Feedback

The clients were asked how much the previous target symptoms had influenced them in a follow-up survey after 1 week.

### Procedure

In this study, a dedicated reservationist establishes a consultation time based on the client's registration information. When the client enters the consultation room, the psychotherapists will spend about 1–3 min gathering basic information and identifying the client's primary symptoms that need to be addressed and a 1–10 rating (i.e., the influence of the clients' target symptoms). The formal consultation session is then conducted, and after the consultation, the 1–10 scale is administered once more. An average single session of ~50 min for each client.

**Figure 1:**
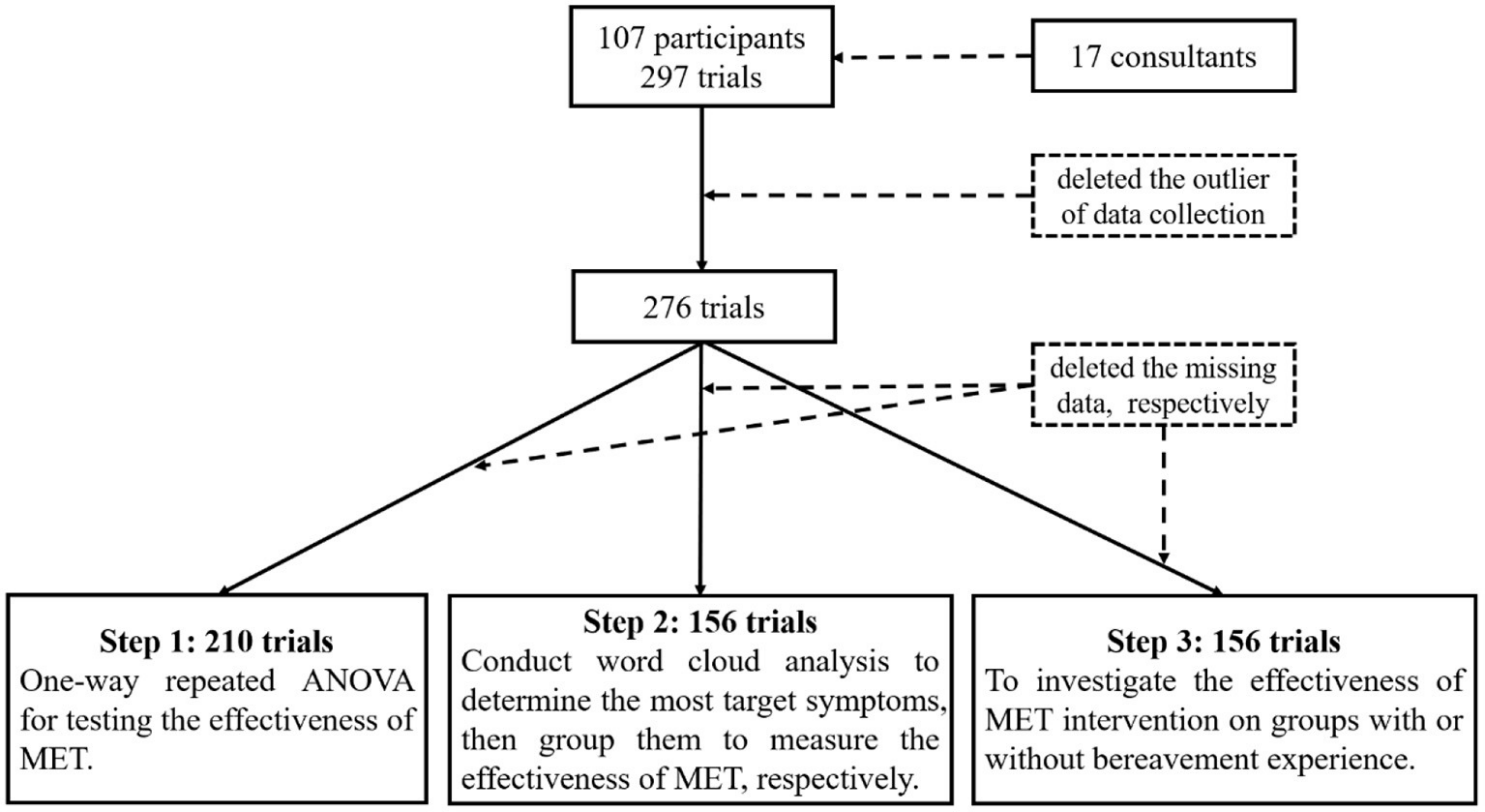
Consort diagram for the present study.

The appointment maker will contact the visitor within about a week to inquire about the consultation's outcome and conduct a 1–10 rating. Due to the principle of respecting client's wishes, data on the effectiveness of counseling are unavailable for some visitors, as shown in Figure 1. In total, 297 sessions were collected. A total of 276 sessions were collected for the final process with outlier deletion (i.e., duplicate and incorrect data rows).

### Statistical Analysis

In the present study, all data were analyzed by R. We used the package of *compareGroups* (9) to collect the essential information for the participants and therapists. Then, the *WordCloud* package (10) was used to visualize the target symptoms of the participants. The *afex* package (11) was used to perform the mixed ANOVA.

## Results

### Descriptive Demographic Analysis

As shown in Tbale 1, we recruited 107 participants, with the majority being female (*n* = 93; 86.9%). More than half of the participants had undergraduate and junior college education degrees (*n* = 69; 64.5%). More than half of them were employed (*n* = 62; 57.9%) and were not students (*n* = 93; 86.9%). More than 60% of them were married (*n* = 69; 64.5%). As reported in the clients' information, most of them did not have mental health issues (*n* = 94; 87.9%) or took medicine (*n* = 90, 84.1%; see Table 1).

**Table 1:** Summary descriptives table for clients (*n* = 107).

	**Variable**	***N* (Proportion/*SD*)**
Gender	Male	14 (13.1%)
	Female	93 (86.9%)
Age		40.0 (10.95)
Education	Below high school	3 (2.80%)
	High school and polytechnic school	3 (2.80%)
	Undergraduate and junior college	69 (64.5%)
	Master and doctor	30 (28.0%)
	Others	2 (1.87%)
Occupation	Worker	4 (3.74%)
	Cadre	10 (9.35%)
	Technician	16 (15.0%)
	Teacher	18 (16.8%)
	Profession	14 (13.1%)
	Others	45 (42.1%)
Job status	Student	11 (10.3%)
	On the job	62 (57.9%)
	Unemployed	13 (12.1%)
	Retire	8 (7.48%)
	Others	13 (12.1%)
Marriage	Unmarried	31 (29.0%)
	Married	69 (64.5%)
	Divorce	4 (3.74%)
	Separation	2 (1.87%)
	Widowed	1 (0.93%)
Students	Yes	14 (13.1%)
	No	93 (86.9%)
Mental health	Yes	13 (12.1%)
	No	94 (87.9%)
Medicine	Yes	17 (15.9%)
	No	90 (84.1%)

A total of 17 psychotherapists worked for this study. Most of them were female (*n* = 15; 88.2%) and aged older than 40 years (*n* = 15; 88.24%). Most of them had an undergraduate education level (*n* = 16; 94.12%). More than half of them had more than 5 years of working experience (*n* = 11; 64.71%). More than half of them had worked in counseling between 1 and 3 years (*n* = 11; 64.7%) and had supervision time below 50 h (*n* = 11; 64.71%; see Table 2).

**Table 2:** Summary descriptives table for psychological consultant (*n* = 17).

**Variable**		***N* (proportion)**
Gender	Female	15 (88.2%)
	Male	2 (11.8%)
Age	20–30	1 (5.88%)
	30–40	1 (5.88%)
	40–50	10 (58.8%)
	50–60	4 (23.5%)
	Above 60	1 (5.88%)
Education	Below undergraduate	1 (5.88%)
	Master	7 (41.2%)
	Undergraduate	9 (52.9%)
Working time	1–3 years	3 (17.6%)
	3–5 years	3 (17.6%)
	5–10 years	6 (35.3%)
	Above 10 years	5 (29.4%)
Treatment time	1–3 years	11 (64.7%)
	Above 5 years	2 (11.8%)
	<1 year	4 (23.5%)
Supervision hours	<20 h	7 (41.2%)
	20–50 h	4 (23.5%)
	Above 50 h	6 (35.3%)

### Descriptive Analysis of the Number of MET Consultations

**Figure 2:**
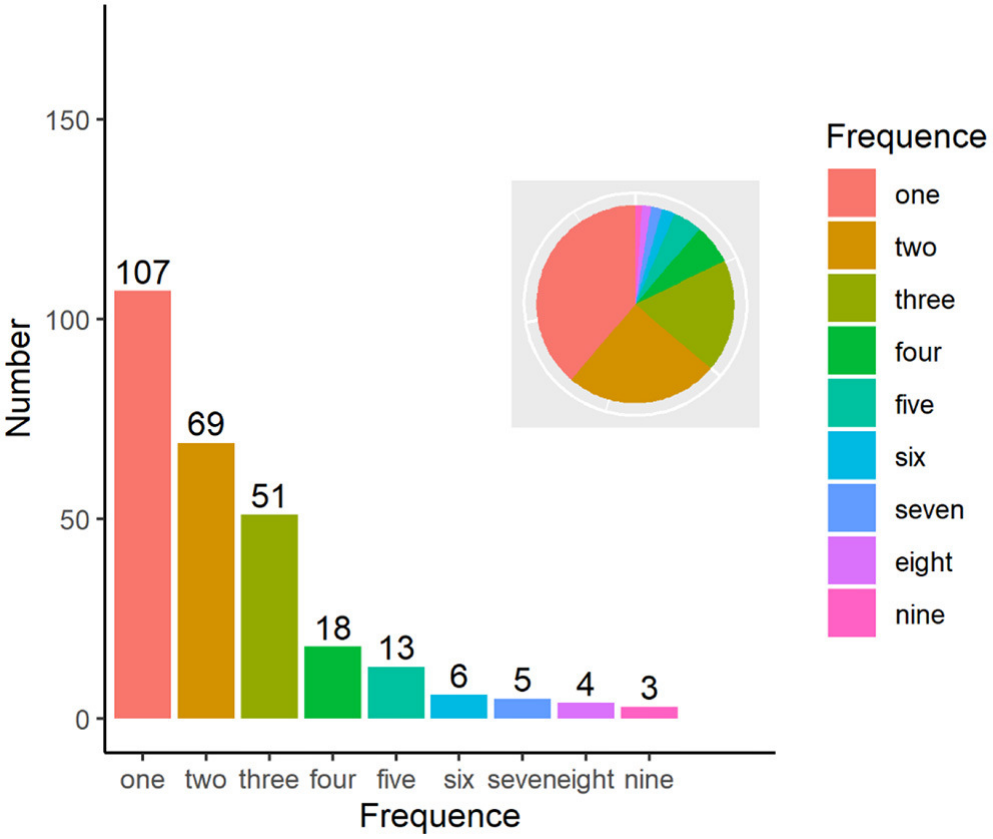
Times of psychological consultation.

A single individual conducted one initial interview and three consultations in the standardized process. In particular, only a few clients required several counseling sessions to achieve the goal. Specifically, a total of 107 people were interviewed and treated first. Sixty-nine people had two sessions, and 51 people consulted three times. As shown in Figure 2, sessions decreased continuously with fewer clients joining subsequent counseling sessions. Three persons took nine sessions, which was the maximum number of sessions for a single person.

### Overall Intervention Effect of MET

**Figure 3:**
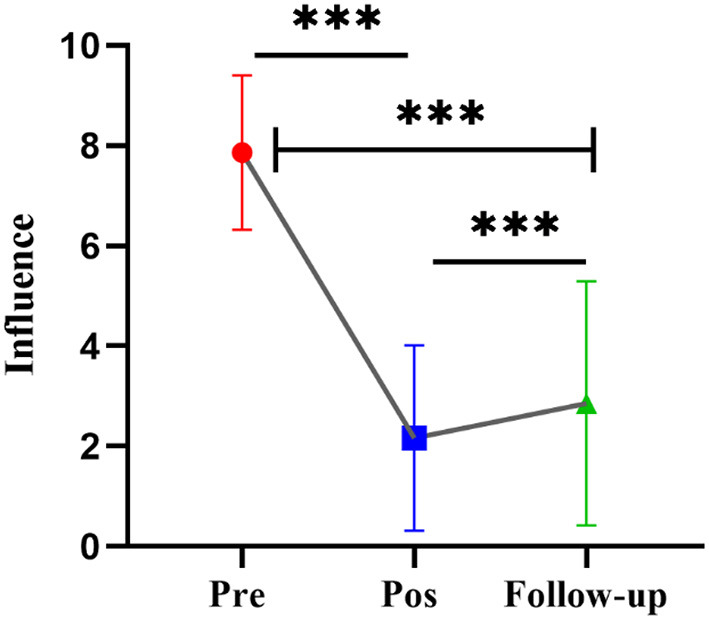
The repeated measurement analysis of influence index. ***p < .001.

Subsequently, this study screened 276 sessions of 107 people who interviewed and consulted for the first time. Only the sessions with consultation participation were retained, and the interview data were deleted if there was no score of the influence index, which included 210 sessions. First, we tested the effectiveness of the MET intervention for participants using one-way repeated-measures ANOVA (Time: Pretest vs. Posttest vs. Follow-up). The results indicated that the main effect of time was significant [*F*_(2, 209)_ = 651.7, *p* = 0.000]. The posttest revealed that the individual influence decreased significantly after the counseling intervention (*Mean* = 2.162) compared with before consultation (*Mean* = 7.861, *t* = 36.08, *p* = 0.000). It should be noted that over time, the influence after follow-up (*Mean* = 2.858) was significantly stronger than that after the intervention (*t* = 4.215, *p* = 0.000), but it was still significantly lower than the influence score before the consultation (*t* = 26.07, *p* = 0.000; see Figure 3).

### Word Cloud Analysis to Categorize Symptoms

**Figure 4:**
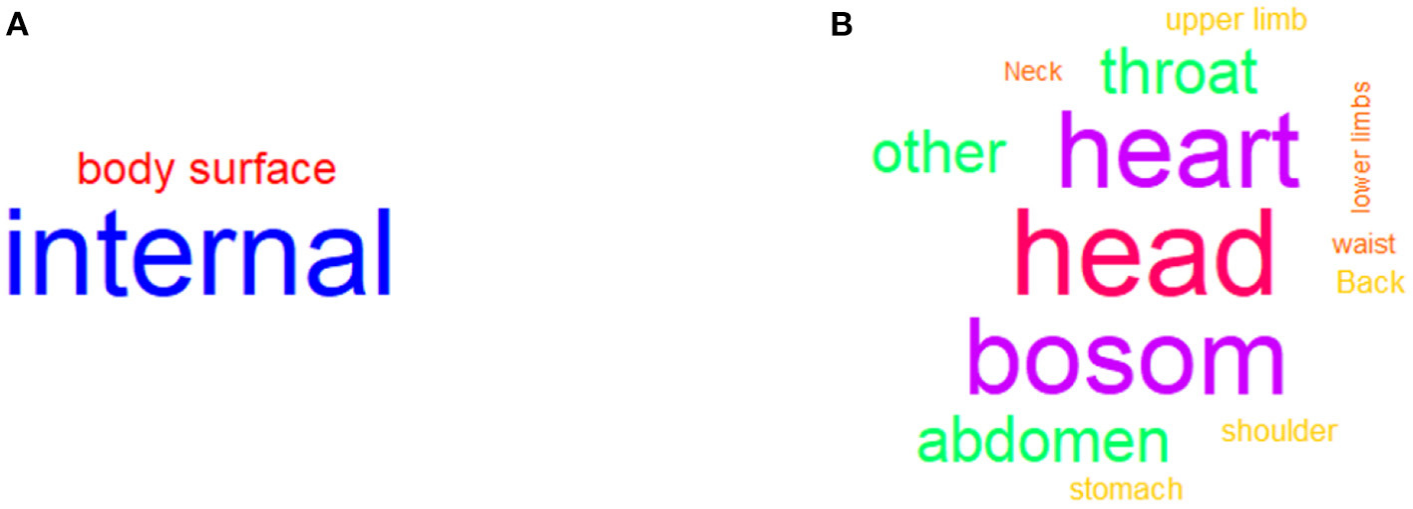
Word cloud analysis of target symptoms. **(A)** indicated the frequency of approximate locations about target symptoms. **(B)** indicated the frequency of specific locations about target symptoms.

Then, we deleted the missing target symptom locations, and 156 sessions were reserved for the word cloud analysis. First, we coded the target symptom locations into two parts: the body surface and internally. The results indicated that 122 symptoms were located inside the body and 34 symptoms were located outside the body (see Figure 4A). Considering the specific location, we coded the target symptoms into 15 parts: head, eyes, throat, neck, shoulders, back, bosom, heart, lungs, waist, stomach, abdomen, upper limbs, lower limbs, and others. The results indicated 30 symptoms for the head, 26 symptoms for the bosom, 26 symptoms for the heart, 14 symptoms for the throat, and 14 symptoms for the abdomen (see Figure 4B).

### ANOVA Analysis for Target Symptoms

Here, it should be pointed out that this study grouped specific locations of target symptoms. The top 3 symptoms (i.e., the three parts of the body where the symptoms appeared the most as the high-frequency group) were defined as the high-frequency group, and the rest were defined as the low-frequency group to explore whether there were differences in the index of influence between the two groups. Furthermore, to investigate the difference in the influence index between the high-frequency and low-frequency groups under three measurements, a mixed ANOVA was conducted: 2 (Group: high-frequency vs. Low-frequency) × 3 (Time: Pretest vs. Posttest vs. Follow up).

The results showed that the main effect of the high-frequency and low-frequency groups was insignificant (*F* = 0.14, *p* = 0.709, η^2^ = 0.001). However, the main effect of the time point was significant (*F* = 461.14, *p* = 0.000, η^2^ = 0.609). The interaction between the high-frequency and low-frequency groups and the time point was significant (*F* = 5.76, *p* = 0.005, η^2^ = 0.019). According to the comparison results, in the low-frequency group, the influence after consultation (*Mean* = 2.20, *SE* = 0.204) was significantly lower than those before consultation (*Mean* = 7.41, *SE* = 0.186, *t* = −19.841, *p* = 0.000) and after follow-up (*Mean* = 3.34, *SE* = 0.283, *t* = −12.440, *p* = 0.000). The influence after follow-up was significantly higher than that after consultation (*t* = 4.403, *p* = 0.002).

**Figure 5:**
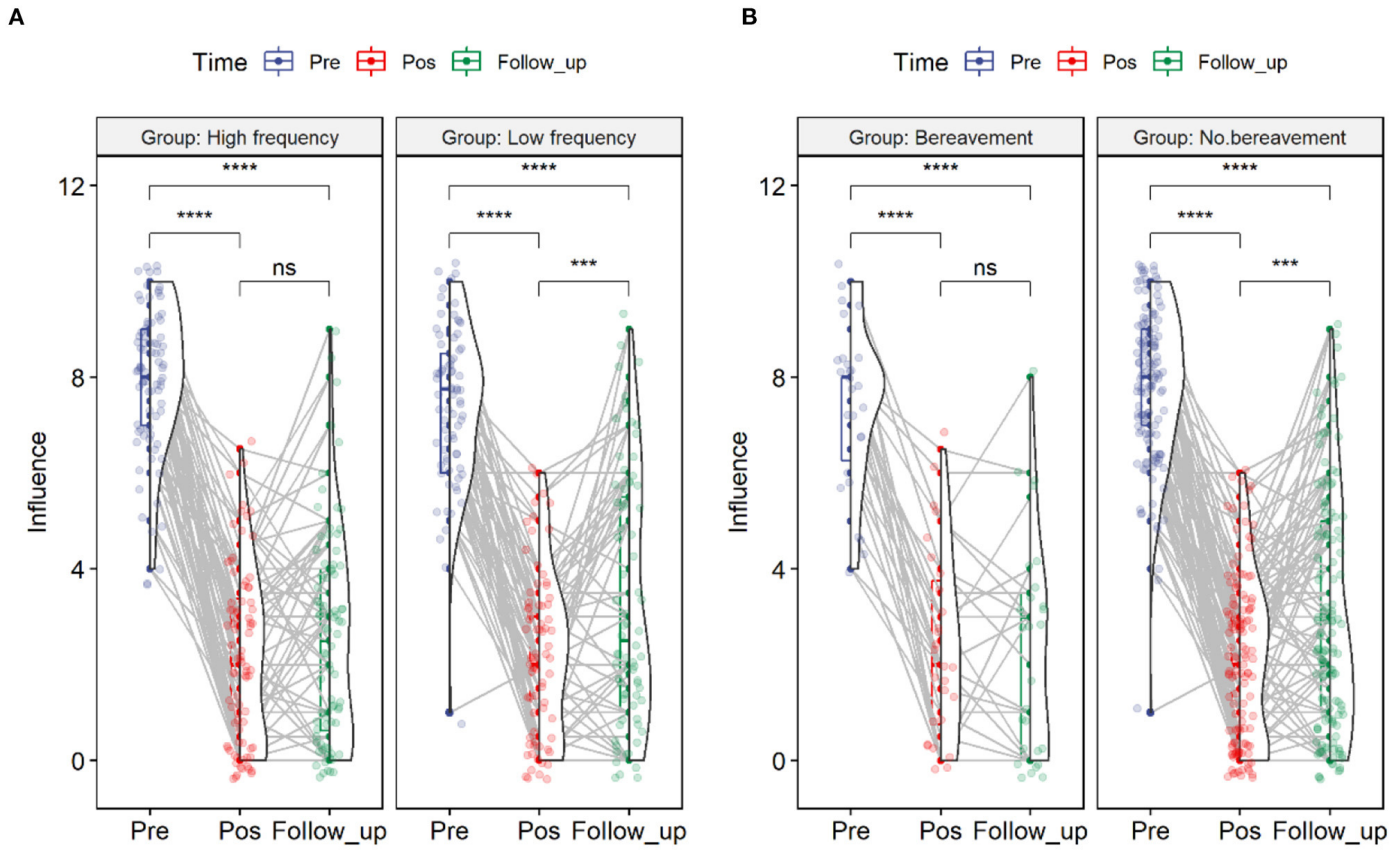
The results of the mixed ANOVA analysis. **(A)** indicated the results of different degrees of frequency. **(B)** showed that whether bereavement or not could have a difference in influence.

In the high-frequency group, the degree of influence after the consultation (*Mean* = 2.16, *SE* = 0.194) was significantly lower than those before consultation (*Mean* = 7.97, *SE* = 0.177, *t* = −23.303, *p* = 0.000) and after follow-up (*Mean* = 2.57, *SE* = 0.269, *t* = 17.384, *p* = 0.000). The degree of influence after follow-up did not significantly differ from that after consultation (*t* = 1.687, *p* = 0.213). The results are shown in Figure 5A.

### ANOVA Analysis for Bereavement

At the same time, after deleting the missing data, 156 sessions were finally obtained. Then, the participants were grouped according to whether they lost relatives during the study time. Furthermore, whether the degree of influence under bereavement was different under the three measurement time points was investigated. That is, we conducted a mixed ANOVA of 2 (Bereavement vs. No Bereavement) X 3 (Pretest vs. Posttest vs. Follow-up). The results showed that the main effect of bereavement was not significant (*F* = 0. 83, *p* = 0.365), and the main effect of the time point was significant (*F* = 252.82, *p* = 0.000), but the interaction between bereavement and the time point was insignificant (*F* = 1.60, *p* = 0.206). According to the comparison results, in the bereavement group, the degree of influence after consultation (*Mean* = 2.39, *SE* = 0.34) was significantly lower than those before consultation (*Mean* = 7.39, *SE* = 0.31, *t* = −11.456, *p* = 0.000) and follow-up (*Mean* = 2.35, *SE* = 0.47, *t* = −9.056, *p* = 0.000). However, there was no significant difference between the degree of influence after follow-up and consultation (*t* = 0.086, *p* = 0.996).

In the non-bereaved group, the degree of influence after consultation (*Mean* = 2.13, *SE* = 0.15) was significantly lower than those before consultation (*Mean* = 7.77, *SE* = 0.14, *t* = −28.261, *p* = 0.000) and after follow-up (*Mean* = 3.01, *SE* = 0.22, *t* = −18.719, *p* = 0.000). The degree of influence after follow-up was significantly lower than that after consultation (*t* = 4.446, *p* = 0.000). The results are shown in Figure 5B.

## Discussion

In summary, the MET preserves somatic relaxation and the CBT operational process while integrating traditional Chinese culture. The MET is highly effective during the COVID-19 pandemic for removing negative emotions and terrible physical feelings. As a safe, fast-acting therapeutic technique, although there is some deterioration after a week, the effects of the MET are mainly maintained, which is essential for bereaved people. Hence, some points are worthy of discussion here.

We recognize that most current counseling techniques in China are derived from the West (12). This provides us with numerous benefits, both essential to those with psychological needs and critical to developing the psychological discipline in China. For different types of clients, divergent therapeutic techniques should be applied. Hence, many people in Eastern cultures can be better served by the MET, which combines the fundamental logic of Western counseling techniques with Eastern philosophical principles.

In the present study, we found that the target symptoms identified by the clients decreased significantly after the intervention. What should not be overlooked is the rebound in the follow-up survey. This also highlights that our consultation outcomes were delivered promptly. According to the follow-up data, the influence of a client's symptoms returns, although it is still significantly below the initial reported levels. It is clear that counseling is pretty successful in improving an individual's current state and that more counseling should be considered progressively over time to strengthen the treatment's benefits.

We separated the top three symptoms according to target symptoms into a high-frequency group and the remainder into a low-frequency group to verify the efficacy of the MET from multiple perspectives. Compared with those in the low-frequency group, there was no significant difference in client effect scores among the high-frequency group at post-intervention and follow-up, which indicates that the effectiveness is apparent when the MET is evaluated from a randomized perspective. The structure of the MET is similar to that of CBT. Nevertheless, the goals are diversified. CBT assists clients in identifying illogical ideas and attempting to adjust their behavior patterns by modifying their irrational or illusory perceptions. The idea of CBT is the circulation among mood, behavior, and cognition. Compared to identifying maladaptive beliefs (13), the MET offers therapists a fresh perspective; it regards people as a whole. The therapeutic target is to remove those severe problems in personalities. CBT is rational, while MET is emotional. In other words, they have different focuses and different treatment goals. The treatment goal of MET is to bring the client to a place where there is no problem. Compared to CBT, MET has short and quick characteristics. Based on the traditional Chinese concept of emptiness, the MET leads clients to dissolve their internal problems into infinite emptiness by identifying and embodying their symptoms.

Bereavement is described as the situation of having lost a significant loved one due to death (14). Everyone will experience bereavement, which is a highly stressful event, during their lifetime. Individuals seldom experience bereavement in early childhood (~3.4%) (15). Then, as people grow, their risks of experiencing bereavement grow, with ~45% of women and 15% of men in elderly populations experiencing bereavement (16). In the present study, participants were grouped according to who had been bereaved and who had not been bereaved. There was no significant difference in the client effect scores among the group with bereavement experiences at post-intervention and follow-up, which indicates that the effectiveness is apparent when the MET is re-evaluated from a randomized perspective.

Bereavement is linked to a higher risk of mortality for a multitude of reasons, including suicide (17). Furthermore, mental and physical illness is severe and persistent in a small proportion of the population. Notably, depending on the individual and their culture, recovery might take months or even years. As a result, bereavement is both a preventative and clinical concern. However, child grief therapies do not appear to produce good results like other professional psychotherapy interventions (18). As children's early intimate relationships come to an abrupt end, early memories fade with aging. Children's bereavement experiences can be reshaped by later social attention and the formation of new personal attachments (19). However, due to the general stability of the object-subject relationship in adulthood (20), adults' memories of painful bereavement experiences are difficult to erase in a short period. They can even remain with them throughout their lives, influencing their daily emotional (21, 22) and life functions (23, 24).

Some researchers have employed a variety of psychological counseling therapies to aid persons experiencing grief who have been bereaved (25–27). Studies investigating counseling techniques refer to the dual process model and the meaning reconstruction model. However, due to cultural differences, the mainstream models in mainland China are currently cognitive behavioral therapy (CBT) and other counseling techniques. To treat and intervene Chinese people who have experienced bereavement, a counseling technique with a high degree of cultural-ecological validity is needed. The moving to the emptiness technique (MET) may be a good choice.

### Limitations

However, this study has potential limitations. To begin, we presently have data on only 107 clients, although these clients reveal great demographic heterogeneity. Given that a large proportion of clients to this study received multiple counseling sessions (see Figure 3), we could not integrate demographic characteristics as covariates in ANOVA analysis. However, in future studies, MET should explore examining the impact of interventions on specific populations and controlling for demographic heterogeneity, such as people with depression. Second, the MET is a new counseling technique that stems from ancient Chinese philosophical thinking and Chinese medical theory, mixed with the logic of Western counseling techniques. Compared with CBT and other techniques, it is still in development. Third, although the therapeutic benefits of the MET were investigated in this study using a randomized design, more analytical approaches, such as questionnaire measurements, fMRI, fNIRS, and other similar methodologies, should be used to assess the efficacy of the MET. Furthermore, the benefits of the theoretical framework of the MET can be used in future studies concerning more clinical patients with specific medical issues, such as neurological headaches and frozen shoulder. The efficacy of the MET may be evaluated by attempting to relieve persons' somatic diseases from a psychological counseling perspective.

## Data Availability Statement

The raw data supporting the conclusions of this article will be made available by the authors, without undue reservation.

## Ethics Statement

The studies involving human participants were reviewed and approved by Beijing University of Chinese Medicine. The patients/participants provided their written informed consent to participate in this study.

## Author Contributions

TL and YC: study design and critical revision of the manuscript. WZ and LL: data collection. YT and YC: analysis and interpretation. YT: drafting of the manuscript. All authors contributed to the article and approved the submitted version.

## Funding

This study was supported by the Scientific Foundation of Institute of Psychology, Chinese Academy of Sciences, No. EOCX331008.

## Conflict of Interest

The authors declare that the research was conducted in the absence of any commercial or financial relationships that could be construed as a potential conflict of interest.

## Publisher's Note

All claims expressed in this article are solely those of the authors and do not necessarily represent those of their affiliated organizations, or those of the publisher, the editors and the reviewers. Any product that may be evaluated in this article, or claim that may be made by its manufacturer, is not guaranteed or endorsed by the publisher.
